# Monitoring the Shelf Life of Hemp Seed Oil Stored at Two Temperatures in Different Materials via Near-Infrared (NIR) Spectroscopy

**DOI:** 10.3390/molecules29235577

**Published:** 2024-11-26

**Authors:** Francesca Bonazza, Lucia Monti, Milena Povolo, Andrea Gasparini, Valeria Pelizzola, Giovanni Cabassi

**Affiliations:** 1 National Research Council, Institute of Sciences of Food Production (ISPA), Via Celoria 2, 20133 Milan, Italy; 2 CREA Research Centre for Animal Production and Aquaculture, Via Antonio Lombardo 11, 26900 Lodi, Italy; lucia.monti@crea.gov.it (L.M.); milena.povolo@crea.gov.it (M.P.); andrea.gasparini@crea.gov.it (A.G.); valeria.pelizzola@crea.gov.it (V.P.); giovanni.cabassi@crea.gov.it (G.C.)

**Keywords:** hempseed oil, shelf life, oxidation, NIR, ASCA model

## Abstract

Hempseed oil (HSO) is extremely rich in unsaturated fatty acids, especially linoleic (18:2 n-6) and α-linolenic (18:3 n-3) acids, which determine its high sensitivity to oxidative and photo-oxidative degradations that can lead to rancidity despite the presence of antioxidant compounds. The aim of this work was to evaluate which material/temperature/light solutions better preserve HSO quality during its shelf life and to test NIR as a rapid, non-destructive technique for monitoring oxidation phenomena. Futura 75 hemp seeds were cold-pressed; the oil was packed into 20 mL vials of four different materials (polypropylene, clear glass, amber glass, and amber glass coated with aluminum foil) and stored for 270 days at 25 °C under diffused light and at 10 °C in dark conditions., Peroxides and conjugated dienes and trienes were evaluated at intervals to monitor oil stability. Moreover, NIR spectra were measured in transmission, and the sample dataset was analyzed using ASCA to test the significance of the experimental factors: the model showed the significance of all factors and of all the simple interactions. Our results demonstrate that oil stored in amber glass vials with aluminum foils at refrigerated temperatures receive the highest protection from environmental conditions, mitigating oxidative changes, and that the NIR technique could be used to rapidly monitor HSO oxidation parameters.

## 1. Introduction

Hemp (*Cannabis sativa* L.) is a very ancient crop that, after a period of abandon, has gained a renewed interest. In recent years, the area dedicated to hemp cultivation for fiber has increased significantly in the EU from 20,540 hectares (ha) in 2015 to 33,020 ha in 2022 (+60%), with an increase in the production of hemp in the same period from 97,130 tons to 179,020 tons (+84.3%) (https://agriculture.ec.europa.eu/farming/crop-productions-and-plant-based-products/hemp_en (URL accessed on 10 September 2024)). Hemp has several environmental benefits, and its cultivation contributes to the achievement of the European Green Deal objectives. Hemp is also a multi-use plant, exploitable in all its forms: stem fibers find applications in the textile, specialty-paper, and construction industries, while sprouts can be used for nutraceutical and/or therapeutic purposes, and seed and essential oils are a valuable resource for green cosmetics, just to list some applications [1,2]. Moreover, worldwide, there continues to be a high level of interest in hemp seeds and their derivative products as a food source. Together with other seed oils, such as canola (*Brassica napus* L.), chia (*Salvia hispanica* L.), and quinoa (*Chenopodium quinoa* Willd.), hempseed oil (HSO) can be considered a functional food due to the presence of several molecules that have positive value for human health [3,4,5]. The principal value of HSO is the high percentage of polyunsaturated fatty acids (PUFAs) (about 80%), especially linoleic (18:2 n-6 at 55 wt%, LA) and α-linolenic (18:3 n-3 at 20 wt%, ALA) acids, which are essential fatty acids (FAs): animals require LA for synthesizing dihomo-γ-linolenic acid (DGLA) and arachidonic acid (AA), which are precursors of prostaglandins. Similarly, ALA must be ingested to produce eicosapentaenoic acid (EPA), essential for the biosynthesis of 3-series prostaglandins [6]. LA plays also a crucial role in maintaining the structural integrity of the skin and its barrier function [7]. In addition, the 3:1 ratio of omega-6/omega-3 in HSO is considered optimal for human health [8,9]. This is because an excess of ALA in the diet can negatively affect metabolic balance, producing a deficit of omega-6 metabolites. Moreover, the presence in HSO of γ-linolenic acid (18:3 n-6, GLA) and stearidonic acid (18:4 n-3, SDA)—two biologic metabolites of LA and ALA in the biosynthesis of prostaglandins—improve the nutritional value of this product. Besides fatty acids, in HSO there are other beneficial components for human health, such as tocopherols, phenolic compounds, carotenoids, sterols, and phytols [10,11]. Tocopherols—bioactive compounds present in HSO in large amounts and in various isomeric forms (α-, β-, γ-, and δ-tocopherol)—vary in concentration depending on hemp varieties, processing methods, and storage conditions. Due to their free-radical-scavenging ability, tocopherols exert a preventive role against degenerative diseases, including cardiovascular issues, Alzheimer’s disease, and certain cancers [10]. The main phenolic compounds in HSO are hydroxycinnamic acid and lignanamides, major plant secondary metabolites; they are produced as defense mechanism against pathogens, reactive oxygen, and UV radiation, thus enhancing the overall antioxidant capacity of oils. Additionally, they also significantly contribute to the organoleptic and nutritional properties of plant-derived foods [5,12,13]. Carotenoids, particularly β-carotene, protect chlorophylls from degradation and prevent color changes during storage [10]. Tocopherols and phenolic compounds, being potent antioxidants, act as “scavengers” against lipid oxidation and play an important role in the chemical stability of oil during its shelf life.

To preserve the high nutritional value and antioxidant compounds characterizing HSO, the cold pressing of seeds is commonly adopted [14,15]. Cold-pressed oils are obtained, without altering the oil, by mechanical procedures only, e.g., expelling or pressing, without the application of heat. In addition, they may be purified by washing with water, settling, filtering, and centrifuging only [16]. In fact, the high amount of PUFAs represents, at the same time, the value and “weakness” of HSO: due to the reactive double bonds among carbon atoms, PUFAs are particularly prone to oxidation. Environmental conditions like oxygen availability, UV light, and temperature are the most important external parameters which can influence both the production process and shelf life. Oxidative and photo-oxidative degradation, occurring through different steps and generating a great number of different molecules, deteriorates the nutritional quality and the sensory perception of the product, reducing its shelf life. During autooxidation, oxygen directly acts on the double bonds of FAs, catalyzed by light, generating radical compounds that start reactions and lead to the formation of primary and secondary oxidation products [17,18,19]. Instead, in the photo-oxidation process, the presence of photosensitizers such as chlorophyll, riboflavin, and heavy metals is required to initiate the oxidation process and produce hydroperoxides [20]. Peroxides and hydroperoxides are primary reaction products; they are odorless and tasteless but highly reactive. They can decompose and react to form a variety of secondary degradation products of different compound classes: aldehydes, ketones, alkanes, alcohols, esters, epoxides, hydroxyl compounds, oligomers, and polymers can form, producing off-flavors in the oil [21] and causing significant changes to its sensory profile.

Given its susceptibility, maintaining the high quality of HSO also throughout its shelf life is essential. Packaging can directly influence the quality of the oil based on the degree of protection it provides against both oxygen and light. Kishimoto [22] reported that extra virgin olive oil stored in clear glass bottles had higher values of free FAs, peroxides, and absorbance values at 270 nm than the same oil stored in covered or amber glass bottles.

To date, there is no specific and harmonized EU legislation for cold-pressed HSO, except for the legal obligation regarding content in terms of the sum of acid and neutral form of D9-tetrahydrocannabinol [23,24]. Not even Codex Alimentarius 210-1999 [16] (amended in 2023), regarding vegetable oils, includes references to HSO. The oil’s oxidative stability is monitored through the determination of the values of acidity, peroxides, and extinctions at 232 nm and 268 nm, which are among the recommended methods of analysis for evaluating the quality of vegetable oils [25]. These techniques, however, are time-consuming, require a certain experience of the operator, and use toxic chemicals. For this reason, there is a need to find other analytical approaches. Spectroscopic techniques are widely investigated as a potential alternative for food analysis and control, having several benefits: they are rapid, are non-destructive, require little to no sample preparation, and are often solvent free. The last aspect is of particular interest in terms of their greenness [26]. Near-infrared (NIR) and mid-infrared spectroscopy (MIR) have been applied to assess the quality and purity of animal and vegetable oil and fats [15,27,28,29] and to determine the geographical origin of vegetable oils [30]. NIR spectroscopy measures the absorption of electromagnetic radiation in the 750–2500 nm (12,000–4550 cm^−1^) range. The analytical signal is a function of the interaction between the sample and the incident radiation. The interaction causes multiple vibrational transitions of the stretching (bond lengthening) and bending (bond angle deformation) present in infrared. The most important absorption bands of the NIR region are related to overtones and combinations of fundamental vibrations of polar functional groups (e.g., OH, CH, and NH), and it is for this reason that NIR spectroscopy is the technique of choice for the characterization of water, lipids, fats, proteins, and carbohydrates. On the other hand, although rapid and green, NIR spectra are not immediately interpretable but should be treated as multivariate data that require the application of chemometric approaches for their interpretation and evaluation.

To the best of our knowledge, no study has been carried out to verify the effects of different packaging materials and storage conditions on the shelf life of HSO simultaneously. Previous works have only dealt with one packaging material and room temperature storage [31] or accelerated storage at 60 °C for a shorter time [21,32]. The aim of our research was to monitor the quality of cold-pressed HSO stored in different conditions, i.e., packaging materials, temperatures, and light exposure, by the application of classical analytical techniques, and to verify the performance of NIR analysis in controlling the oil’s shelf-life. Analysis of Variance–Simultaneous Component Analysis (ASCA) [33] was applied to NIR spectra to evaluate the influence of experimental factors (time, temperature, and storage materials and their interactions) on the physico-chemical phenomena taking place during HSO shelf life and to investigate whether they have a statistical effect on variation in collected spectral data.

## 2. Results and Discussion

### 2.1. Chemical Composition of HSO

**Figure 1:**
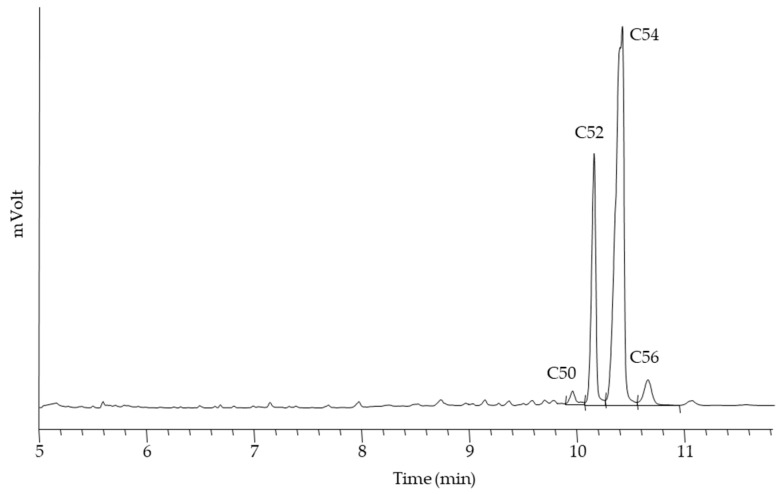
GC profile of triacylglycerols in HSO. The peak labels correspond to the total number of carbon atoms of the FAs esterified in the molecule, excluding glycerol.

HSO was characterized for triglycerides (TAGs), fatty acids (FAs), total carotenoids, and chlorophyll composition. TAGs were separated and identified on the basis of the total number of carbon atoms in the FAs esterified in the molecule, excluding glycerol (Figure 1).

**Figure 2:**
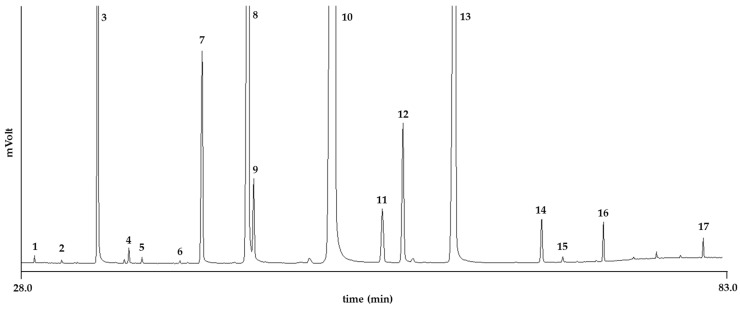
GC profile of fatty acid methyl esters of HSO. Peaks are numbered as in Table 1.

The results obtained showed a predominance of C54 (72.19 ± 2.57%), followed by C52 (23.12 ± 2.10%), C56 (3.73 ± 0.29%), and C50 (0.95 ± 0.17%), consistent with the high content of C18 fatty acids. The data obtained for the FAs’ composition (Figure 2, Table 1) are comparable with those reported by other authors and fall within the range of variability present in the literature [8,34,35,36].

The content of chlorophyll (25.9 ± 0.8 mg kg^−1^) detected in the HSO used in our research is within the range reported by other authors [3,11,37,38]. Large differences in the content of chlorophyll in HSO have been reported. Having pro-oxidant and photosensitizer effects [17], its presence can affect the oxidative stability of HSO. For this reason, some processes can be applied to reduce its content in the final product (e.g., refining and bleaching). No process was applied in this sense on our oil, apart from natural decanting. Also, with regard to the quantity of carotenoids, a high variability is present in the literature data [39]. The value found in our sample (5.4 ± 0.2 mg kg^−1^) is higher than that reported by Izzo et al. [11] but lower than that of other authors [36,37].

### 2.2. Oil Oxidation During Shelf Life

Oxidation is the most dominant chemical process which takes place during oil shelf life, influenced especially by oxygen availability, UV light, and temperature. In order to evaluate the best storage conditions of HSO and the effect of the different variables on its stability, cold-pressed HSO was packaged in vials made of four different types of materials and kept at two storage conditions; as for temperature and light, the HSO was left in the dark at 10 °C and under diffused light at 25 °C. Peroxide values (PVs) and absorbance values at 232 and 268 nm were monitored during the HSO’s shelf life.

**Figure 3:**
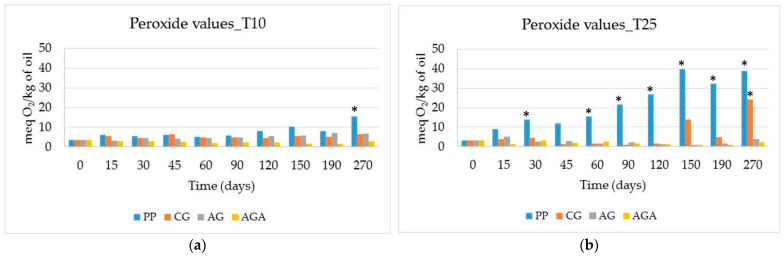
Trends in peroxide content during the storage period at 10 °C (**a**) and 25 °C (**b**). Vial materials: PP = polypropylene, CG = clear glass; AG = amber glass; AGA = amber glass covered with aluminum foil. ANOVA: * *p* < 0.05.

Figure 3 illustrates the peroxide content behavior of HSO packaged in polypropylene, clear glass, amber glass, and amber glass with aluminum foil vials over the storage period at 10 °C and 25 °C.

The PV obtained by iodometric titration is a measure of peroxide and hydroperoxide formation during the initial stages of lipid oxidation, and consequently, it is a good indicator of the amount of primary oxidation products in fresh oils. In general, the PV is low in fresh good oil and increases as the oxidation process goes on. In addition, the PV decreases as secondary oxidation products appear, so the PV may also be relatively low in rancid oil, and its measure should be associated with other parameters related to the development of secondary oxidation products.

In the absence of specific regulation, the limit of 15 meq O_2_/kg oil reported in Codex Alimentarius for cold-pressed virgin oils not covered by individual standards can also be considered for HSO [40]. In addition, the EU Regulation setting marketing standards for olive oil [24] could be taken into account; however, this directive fixes a maximum value of 20 meq O_2_/kg oil for extra virgin olive oil (EVOO), which is even higher than the limit imposed by Codex Standard.

Considering the most stringent threshold value, samples stored at 10 °C in the dark exhibited only a limited increase in the PV in all packages, with values < 10 meq O_2_/kg oil. In dark conditions, only autoxidative processes take place, so it is only oxygen that is responsible and triggers a free-radical chain reaction. In fact, in these experimental conditions, only samples stored in polypropylene, which is partially permeable to oxygen, had a drastic increase at the end of their shelf life and reached the limit after more than 200 days of storage (15.41 meq O_2_/kg oil at 270 days). On the contrary, glass vials represented a better barrier against oxygen permeation and protected oil from oxidation.

Conversely, at 25 °C under diffused light, both autoxidation and photo-oxidation are responsible for the oxidative processes, with an additive effect. Under these conditions, only amber glass vials with aluminum foil protected the oil, with no increase in the PV, and the values were in line with those registered during storage at 10 °C. On the other side, polypropylene vials had the worst performance, approaching the limit of 15 meq O_2_/kg oil starting from the first 30 days of storage and reaching values of 40 meq O_2_/kg oil at 150 days. Under these conditions, multiple factors influenced the process: higher temperature accelerated the free-radical reaction started by oxygen, and light increased oil oxidation, favored by the presence of chlorophyll, which acts as a pro-oxidant.

Glass vials, both clear and amber without aluminum foil, showed intermediate behavior, with fluctuations. For example, in HSO kept in clear glass, after 120 days of storage, a significant increase in peroxide value was observed; however, in oil packaged in clear and amber glass, after period of 45–120 days, the PV was even lower than the values detected at 10 °C. The different kinetics of oxidative reactions at the two temperatures can be a possible explanation of these results. At 25 °C, once oxygen is consumed, the secondary oxidation reactions proceed more rapidly than at 10 °C, thus determining a faster reduction of the PVs and an increase in secondary compounds.

Amber glass bottles gave good results in the work of Tura et al. [31] as well; the authors packaged HSO samples in amber glass bottles and stored them for three months at ambient temperature with a dark–light cycle of 12 h each and recorded the PVs. Peroxides, starting from 2.66 ± 0.29 mEq O_2_/kg of oil at the beginning of shelf life, showed fluctuations but did not increase during the 3 months of storage and the PVs remained lower than the maximum limit fixed by Codex Standard 19-1981 [40].

Other authors have screened the quality indexes and composition of HSO purchased from supermarkets and consequently stored the oil under unknown and variable conditions. They found PVs in HSO samples ranging across a wide interval: Tura et al. [39] found PVs from 3.97 to 11.93, with one sample exceeding to 23.89 meq O_2_/kg of oil. Spano et al. [41] studied nine commercial HSO samples from different countries and found great variability in the PVs, ranging from 4.32 to 22.14 meq O_2_/kg. Piskernik et al. [42] determined a PV greater than 15 meq O_2_/kg for all of the HSO samples they analyzed, ranging from 23.7 to 77.2.

Combined with PVs, UV spectrophotometric analysis gives a measure of the degree of oxidation of oil. Spectrophotometry is a simple and efficient technique for evaluating their quality by measuring the absorbances of oils dissolved in certain solvents at specific UV wavelengths. In general, good-quality oils with a high content of polyunsaturated fatty acids have low absorption in the spectral range of 200 to 300 nm but during some industrial processes or as a consequence of aging or bad storage, UV-absorbing compounds can develop. Hydroperoxide formation can be accompanied by the stabilization of the radical state via double-bond rearrangement (electron delocalization), leading to the formation of two (dienes) or three (trienes) conjugated double bonds that absorb UV light at 232 and 268 nm, respectively. Adsorption at 232 nm is attributed to primary oxidation products developing during the initial phases of HSO oxidation, while at 268 nm, secondary oxidation products, mainly aldehydes and ketones, also absorb UV light. During HSO shelf-life analysis, UV data, read as absorbance values, were expressed as extinction coefficients (K_232_ and K_268_), which represent the specific adsorptivity values of a 1% solution of oil in isooctane at the two specific wavelengths.

**Figure 4:**
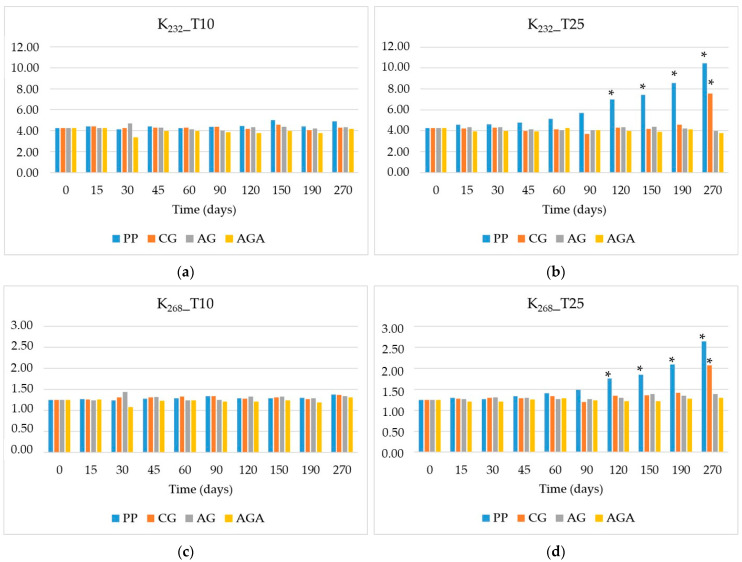
Trends in diene (K_232_) content at 10 °C (**a**) and 25 °C (**b**) and triene (K_268_) content at 10 °C (**c**) and 25 °C (**d**), during the storage period. Vial materials: PP = polypropylene, CG = clear glass; AG = amber glass; AGA = amber glass covered with aluminum foil. ANOVA: * *p* < 0.05.

Figure 4 shows the trends in diene and triene content over the storage period at 10 and 25 °C. As it was for the peroxide values, no specific regulation has been reported for diene and triene content in HSO, and also, no reference values are fixed in Codex 19-1981 [40] for cold-pressed virgin oils not covered by individual standards. The only values we can refer to are the threshold values established by EU Regulation setting marketing standards for olive oil [25], which fixes K_232_ ≤ 2.50 and K_268_ ≤ 0.22 for EVOO. These values are by far lower than the results we obtained for HSO in the present work: starting from time zero, higher values of K_232_ and K_268_ were detected (K_232_ = 4.26 and K_268_ = 1.25). It is likely that, even during the pressing phase, the temperatures reached in the press, in the presence of oxygen, may induce a certain level of oxidation in the HSO. The oil produced for the experiment was obtained from a small batch of seeds using a small-scale press, which may have influenced the oxidative processes observed during the extraction [37]. In addition, the behavior of diene conjugation agreed well with the peroxide values. Similarly to the peroxide content, not only the diene but also the triene levels in samples stored in polypropylene rose more sharply compared to those stored in glass materials: in samples stored at 25 °C, K_232_ and K_268_ increased up to 10.44 and 2.64, respectively. On the contrary, the barrier effect of glass limited oxygen permeation, thereby reducing the formation of these oxidative products, even though clear glass was performing less well and allowed an increase in dienes and trienes at 25 °C in the last two months of storage (K_232_ = 7.55 and K_268_ = 2.07 at 270 days of storage). Notably, the samples in amber glass with aluminum foil consistently demonstrated the lowest levels of dienes and trienes, underscoring the effectiveness of this material in preserving oil quality by mitigating photo-oxidative degradation. Our results are in line with the data of Anwar et al. [43], who characterized HSOs from different agro-ecological zones of Pakistan and found specific extinctions at 232 and 270 nm ranging from 3.50 to 4.18 and from 0.95 to 1.43, respectively. In addition, a high range of values for diene and triene content in HSO has been reported in the literature. For example, Spano et al. [41] characterized nine samples of commercial HSO: the K_232_ value turned out to be generally below the limit reported for EVOO (ranging from 1.72 to 2.65), whereas the K_270_ value was higher (range from 0.19 to 0.69); however, both of these values were lower than those of our results. The higher conjugation of triene content was attributed to the higher amount of PUFAs and, specifically, of triunsaturated fatty chains in HSO with respect to olive oils. Also, Tura et al. [31] monitored the evolution of UV absorbances in HSO over a period of 3 months of storage at ambient temperature and found that the difference between the initial and final values of K_232_ and K_268_ was not statistically significant: around 2.5 and 0.45, respectively. Conversely, after 10 days of storage, the values unexpectedly increased (3.82 ± 0.62 and 0.73 ± 0.07, respectively), approaching our values.

### 2.3. NIR Spectra Acquisition

**Figure 5:**
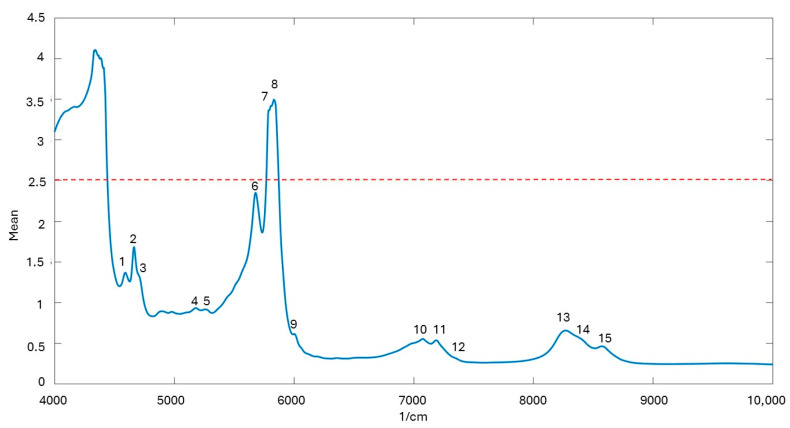
Mean NIR spectrum of the analyzed HSO samples. Numbers refer to specific wavenumbers, whose assignments are reported in Table 2. Dashed line at 2.5 absorbance units: threshold beyond which spectral data were discarded during the processing of data because of saturation effects.

In Figure 5, a mean spectrum of the analyzed HSOs is reported. Due to the elevated optical path (4 mm), the spectral bands in the region from 4000 to 4440 cm⁻^1^ and between 5770 and 5875 cm⁻^1^ exhibited signal saturation with absorbance values exceeding 2.5. The dashed line indicates the threshold of 2.5 absorbance units, beyond which the spectral data were discarded to maintain linearity in the response for subsequent analyses. Table 2 provides the detailed assignment of the main spectral bands observed in the NIR spectrum of HSO. The table highlights key vibrational transitions, including C-H stretching and bending, C=C stretching combinations, and overtones of the C=O stretch. These bands are essential for characterizing the molecular interactions within the oil, particularly those associated with the functional groups of lipids and hydrocarbons. Notably, the presence of bands related to methylenic and methyl groups (CH_2_ and CH_3_), as well as specific cis C=C stretching vibrations, underscores the complex unsaturated FA composition of HSO. The band at 5269 cm^−1^ (water combination) is probably due to the presence of water bounded to unsaponifiable fraction of HSO.

### 2.4. ASCA Analysis of Spectral Variation

The Analysis of Variance–Simultaneous Component Analysis (ASCA) is a statistical technique used to analyze complex datasets involving both categorical and continuous variables [44]. This approach combines Analysis of Variance (ANOVA) and Principal Component Analysis (PCA). Initially, ANOVA decomposes the data into main effects and interactions among experimental factors, isolating the variability attributable to each factor. Subsequently, PCA is applied to reduce the dimensionality of the data and identify the principal components that explain the residual variance. This method allows for the assessment of the statistical significance of factors and their interactions in the context of complex experiments, such as the study of HSO stability under various storage conditions. In this study, ASCA enabled the determination of the significant effects of storage time, temperature, and packaging material on HSO quality, highlighting the key interactions between these factors.

The sample dataset of spectra was analyzed using ASCA to test the significance of the experimental factors, according to the following model:

**X** = **μ** + **X**time + **X**temp + **X**mat + **X**time x temp + **X**temp x mat + **X**mat x time + **E** (1)

where **μ** represents the vector of the mean response, i.e., the mean spectrum of the oil dataset.

ASCA analysis on spectral data typically involves the use of empirical probability distributions rather than relying on theoretical distributions, allowing for a more data-driven approach that accounts for the specific characteristics of the experimental dataset. An IA permutation test where the y-block is shuffled allows for the calculation of the probability that the results obtained with the unperturbed y-block are significant (as compared to random chance). In this work, the analysis of the ASCA model was tested using 5000 random permutations of spectra with respect to experimental matrix and showed significance with a confidence of 5% of all factors and of all simple interactions. From Table 3, it can be inferred that the main effect is due to the storage time. Among the interactions, as expected, the ones between storage time and material and between time and temperature showed a greater effect.

**Figure 6:**
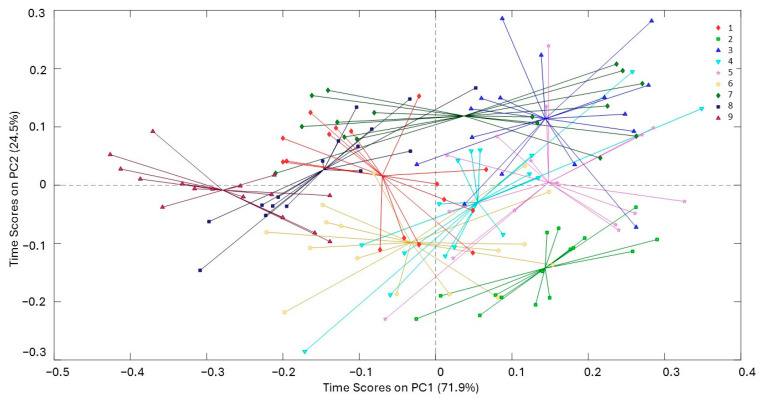
Spider score plots of PC1 for the time factor (1 = 15 days, 9 = 270 days; see Materials and Methods Section for the attribution of all times); explained variability, PC1 = 71.9% and PC2 = 24.5%.

**Figure 7:**
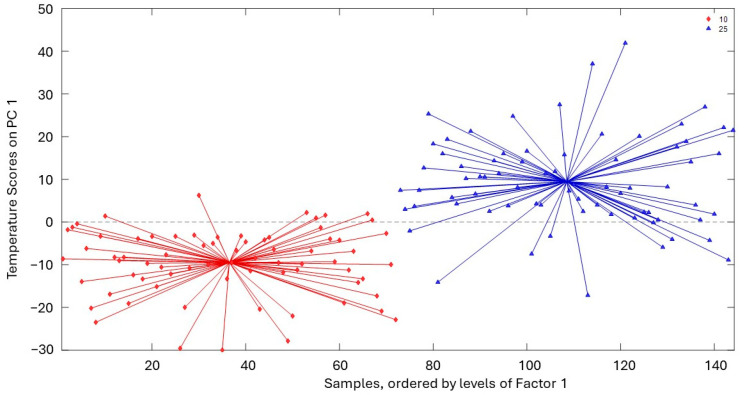
Spider score plots of PC1 for the temperature factor (10 °C and 25 °C).

**Figure 8:**
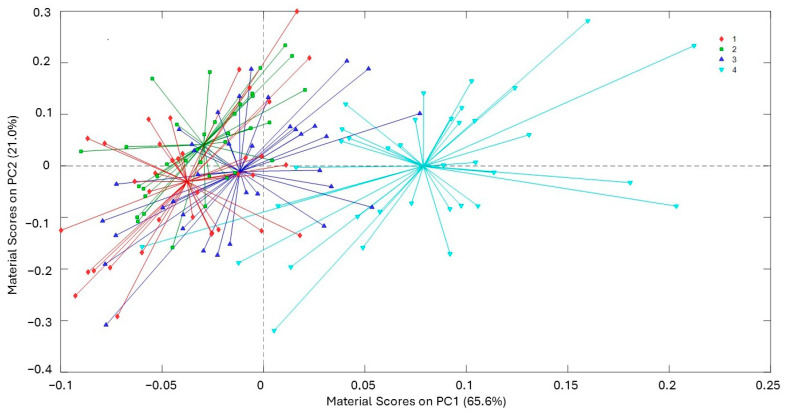
Spider score plots of PC1 for the material factor (1 = polypropylene, 2 = clear glass, 3 = amber glass, 4 = amber glass with aluminum foil). Explained variability, PC1 = 65.6% and PC2 = 21.0%.

The analysis of the curves presented in Figure 6, Figure 7 and Figure 8 reveals significant insights into the stability and degradation of HSO under different storage conditions.

Figure 6 illustrates the spider score plots of PC1 and PC2 for the time factor, demonstrating that the samples’ spectral characteristics change notably over the storage period, with complex behavior around the starting time (time 1). It is noteworthy that the clusters for the data corresponding to times 1–6 exhibit greater radial symmetry around the centroid, whereas for the later time points, an elliptical dispersion becomes evident.

Figure 7 displays the spider score plots of PC1 (explaining 100% of variability) for the temperature factor, highlighting the combined effects of temperature and light conditions on HSO stability. The separation of data points corresponding to different temperatures indicates that storage temperature significantly influences the rate of oxidative degradation, with higher temperatures accelerating the process.

In Figure 8, the spider score plots of PC1 and PC2 for the material factor is presented: the plot shows that HSO samples stored in amber glass with aluminum foil are consistently distinguishable from those stored in other materials. This suggests that this packaging solution effectively protects oil from environmental conditions and mitigates oxidative changes, thereby preserving the oil’s quality better than other materials.

**Figure 9:**
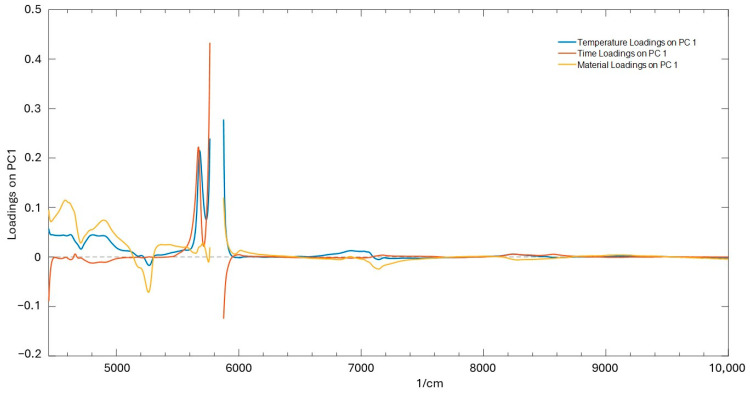
Loading plot for PC1 of time (red line), temperature (blue line), and material (yellow line) factors.

Figure 9 displays the overlay of the PC1 loadings for the three experimental factors (time, temperature, and material). The use of minimal data preprocessing (baseline correction applied to remove residual turbidity effects and centering on the mean) allowed for the maximization of the interpretability of the models. The PC1 loading for the “temperature” factor shows the significant weight of the symmetric and asymmetric second overtone signals of the methylenic groups in the aliphatic chains at 5678 cm⁻^1^ and 5892 cm⁻^1^, respectively. Similarly, in the case of the PC1 loadings, for the “time” factor, the most important bands are the same, but with a positive sign for the lower wavenumber band and a negative sign for the higher wavenumber band. In the case of the “material” factor, however, the greatest weight is given by signals attributed to the vibrations of double bonds between 4600 and 5180 cm⁻^1^. For the “temperature” factor, the strong loading of both symmetric and asymmetric methylenic group overtones indicates that temperature affects the integrity and conformation of the aliphatic chains in the fatty acids. Higher temperatures likely accelerate molecular motion and promote oxidation, leading to changes in these vibrational modes [45].

Regarding the “time” factor, the shift in the loading sign between the two methylenic group bands may indicate a progression in the oxidative state of the oil. The positive loading for the lower wavenumber band (5678 cm⁻^1^) and the negative loading for the higher wavenumber band (5892 cm⁻^1^) might represent a later stage of degradation, where more extensive breakdown of the aliphatic chains has occurred, resulting in different vibrational signatures.

The spectral dataset was also used to test the predictive ability of NIR analysis with respect to parameters that measure HSO degradation. In fact, the three-way ANOVA for all three chemical degradation parameters of the oil analyzed demonstrated the significance of all factors considered in the experimental design, as well as the significance of their interactions. The ability of the NIR technique to discriminate the early rancidity of oil was highlighted, with satisfactory performance for the PLS models developed for the number of peroxides, K_232_ and K_268_. The prediction errors in cross-validation (Rmsecv) (excluding a “material” group at a time as a cancellation subset) were 2.50, 0.57, and 0.12, respectively, corresponding to RPDs (Ratios of Performance to Deviation) of 3.04, 1.51, and 1.83, respectively (Table 4).

**Figure 10:**
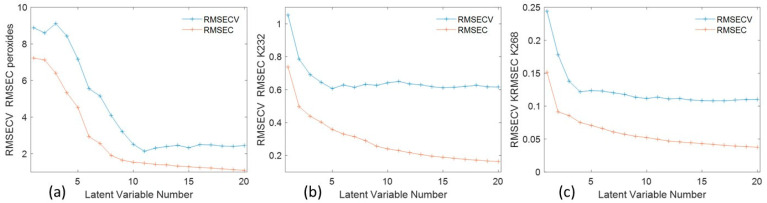
Graphs of Rmsec and Rmsecv as a function of latent variables for peroxides (**a**), dienes (**b**), and trienes (**c**).

In the calibration model, latent variables (LVs) are linear combinations of the original variables, designed to capture the maximum variance while reducing the dimensionality of the dataset. In PLS regression, they are designed to maximize the shared variance (covariance) between the predictor and response matrices. In Figure 10, graphs of Rmsec and Rmsecv as a function of latent variables are presented for peroxides and dienes (K_232_) and trienes (K_268_). Bias represents the systematic difference between the predicted values and the true value that was estimated as the average error over all predictions.

## 3. Materials and Methods

### 3.1. Materials

Hemp seed oil of Futura 75 obtained via the cold-pressing of seeds was supplied by NextFarm S.r.l. (Bagnolo Cremasco (CR) Italy). After pressing, the oil was stored in an aluminum container and delivered to the laboratory the next day. Afterwards, the oil was left to rest for 2 weeks, in order to allow its sediments to settle. The oil was then packed into 20 mL vials made of different materials: polypropylene, clear glass, amber glass, and amber glass coated with aluminum foil. The vials were filled to the top to reduce the head space and limit the presence of oxygen, which would favor oxidative phenomena. The samples were stored for 270 days at ambient temperature under diffused light (T 25 °C) and at refrigeration temperature in dark conditions (T 10 °C) to simulate two common storage conditions, that is, exposure on a shelf in a supermarket and domestic storage, respectively. In order to mimic retailer conditions, the temperature 25 °C was kept constant by an air conditioning system working 24 h/24 h, while diffused light followed natural sun/dark cycles from a window. To reproduce refrigeration conditions, the samples were placed in a refrigerator at 10 °C, equipped with a thermometer to check the temperature daily, and dark conditions were ensured by closing the refrigerator door: in this way, the protection from light was constant, apart from the moment of opening the door to remove the vials for analysis.

The samples were then analyzed at fixed times during their shelf life. For each condition, two vials were analyzed, and analyses were performed in duplicate.

The experimental conditions are reported in Table 5.

All the chemicals used were of analytical grade unless stated otherwise.

### 3.2. Triacylglycerol Composition

The analysis was performed following Povolo et al. [46]. An Easy 1 (Agilent Technologies, Palo Alto, CA, USA) capillary column (4 m length, 0.32 mm i.d., 0.1 μm film thickness) and a flame-ionization detector at 350 °C were used. A fat sample was dissolved in hexane (concentration of 3 mg/mL), and 1 μL was injected via on-column injection. Hydrogen was used as a carrier gas at a flow rate of 5 mL/min. The oven temperature was held at 60 °C for 2 min, programmed to 340 °C at a rate of 35 °C min^−1^, and held at 340 °C for 5 min. Triglycerides were identified on the basis of the total number of carbon atoms, excluding glycerol, by injecting commercial standards, when available. The results are expressed as mass fractions (%).

### 3.3. Fatty Acid Composition

Fatty acids were determined as methyl esters (FAMEs), prepared by the base-catalyzed methanolysis of glycerides using KOH 2N in methanol [47]. The FAMEs were analyzed with a TraceGC (ThermoFisher, Rodano, Milan, Italy) gas chromatograph, equipped with a CP-Sil 88 (Varian, Santa Clara, CA, USA) capillary column (100 m length, 0.25 mm i.d., 0.20 μm film thickness) and a flame ionization detector (FID) maintained at 250 °C. The injection was performed using a PTV injector in split mode (split ratio 1:100), at a constant temperature of 250 °C and using hydrogen (0.5 mL min^−1^) as a carrier gas. The temperature conditions were as follows: 45 °C for 8 min, programmed to 173 °C at a rate of 12 °C min^−1^, held at 173 °C for 47 min, programmed to 220 °C at a rate of 4 °C min^−1^, and held at 220 °C for 20 min. Individual fatty acid methyl esters were identified via comparisons to the standard mixture Nu-Chek GLC-463 (Nu-Chek Prep Inc., Elysian, MN, USA) and pure standard of stearidonic acid methyl ester (Sigma-Aldrich, St. Louis, MO, USA).

### 3.4. Chlorophyll and Carotenoid Content

Chlorophyll and carotenoid content was determined by UV spectroscopy adapted from the protocol used in [48]. Briefly, 0.1 g of HSO was diluted with a 1.5 mL solution of hexane/ethyl acetate/acetone (2:1:1) and 1% butylated hydroxytoluene (BHT) solution in methanol as an antioxidant. Then, the extract was vortexed, sonicated for 10 min, and centrifuged at 10,000× *g* rpm at 4 °C for 10 min. Afterwards, 900 μL of the solution was transferred into a quartz cuvette and scanned in the range 380–700 nm in a dual-beam spectrophotometer (Jasco V-530, Jasco Europe S.r.l. Cremella (LC) Italy). Absorbances at 428 and 661 nm for chlorophyll a and 453 and 642 nm for chlorophyll b were read, in addition to 470 nm for carotenoids. The concentration of pigments was calculated by applying equations reported in the aforementioned protocol.

### 3.5. Peroxide Values

The peroxide number (expressed as meq O_2_/kg of oil) was determined using the iodometric technique, according to ISO 3960:2017 [49]. Depending on the estimated content of peroxides, an aliquot of oil ranging from 2 to 5 g was dissolved in chloroform, acetic acid, and potassium iodide saturated in aqueous solution. After 1 min of shaking and 5 min of rest under protection from light, distilled water was added, and the solution was titrated with a sodium thiosulfate solution, using a starch solution as indicator, and was vigorously shaken. The reaction was complete when the color of the solution changed from brown/violet to yellow/orange. The peroxide number was calculated as the product of the volume of titrant expressed in ml, with the normality of the titrant, divided by the weight of the sample expressed in kg.

### 3.6. Spectrophotometric Investigation in the Ultraviolet Range

Spectrophotometric analysis was used to measure the conjugated dienes and trienes of the polyunsaturated fatty acids formed during the storage period, according to ISO 3656:2011 [50]. Briefly, 0.25 g of oil was dissolved in 50 mL of iso-octane solvent, and the absorbances were measured in a dual-beam spectrophotometer (Jasco V-530, Jasco Europe S.r.l. Cremella (LC) Italy), at 232 nm and 268 nm, with respect to the pure solvent, in 10 mm optical path quartz cuvettes.

The specific extinction values K_232_ and K_268_ were calculated as a ratio between the absorbance at 232 and 268 nm, respectively; the product of the concentration of the solution was calculated in g/100 mL; and the path length of the quartz cell was calculated in cm. The result is expressed to two decimal places.

### 3.7. Spectral Acquisition

NIR spectra were measured in transmission mode using an FT-NIR NIRFlex N-500 spectrometer equipped with NIRWare instrument control software version 1.5 (BÜCHI Labortechnik AG, Flawil, Switzerland). The samples were transferred to cylindrical glass cuvettes with an optical path length of 4 mm and were heated at 40 °C in a dry-block heater (MPM Instruments S.r.l., Bernareggio (MB) Italy).

### 3.8. Statistics

ASCA (ANOVA–Simultaneous Component Analysis) and NIR calibration models were developed using PLS toolbox 9.3 (Egenvector Research, Inc., Manson, WA USA) running under Matlab (2022b) (The Mathworks).

Three-way ANOVA with fixed factors and post hoc analysis (Tukey test) were performed and PLS regression models were used through the Matlab Statistics and Machine Learning Toolbox v 12.4.

## 4. Conclusions

The trends depicted by the PV and spectrophotometric analysis provided a comprehensive overview of the oxidative stability of HSO under various storage conditions. The data showed that samples stored in amber glass covered with aluminum foil develop only minimal amounts of peroxides, indicating superior protection of this kind of packaging against oxidation in both temperature/light conditions.

The ASCA analysis highlighted the significant impact of storage time on the oxidative stability of HSO. The interactions between storage time and material and between storage time and temperature were particularly noteworthy. These results demonstrated the protective effect of materials that offer maximum isolation from both light and oxygen against oxidation over time and the detrimental effect of higher temperatures. Future studies could explore the molecular mechanisms underlying these interactions to develop more effective packaging solutions for HSO and similar products.

This work also pointed out the ability of NIR to discriminate the early rancidity of oil due to the effects of oxidation and that NIR can be applied as a rapid technique for fast monitoring of HSO quality.

## Figures and Tables

**Table 1:** Composition of fatty acids (Σ-SFA, sum of saturated fatty acids; Σ-MUFA, sum of monounsaturated fatty acids; Σ-PUFA, sum of polyunsaturated fatty acids) of HSO.

Peak Number	Component (% Total FAME)	Fatty Acids	
		Mean	SD
1	Myristic, C14:0	0.03	0.000
2	Pentadecanoic, C15:0	0.01	0.001
3	Palmitic, C16:0	6.50	0.014
4	Palmitoleic, C16:1 *c*9	0.10	0.001
5	Eptadecanoic, C17:0	0.04	0.001
6	Eptadecenoic, C17:1 *c*9	0.02	0.001
7	Stearic, C18:0	2.77	0.011
8	Oleic, C18:1 *c*9	13.12	0.010
9	Octadecenoic, C18:1 *c*11	1.02	0.035
10	Linoleic, C18:2 n-6	56.77	0.021
11	Arachidic, C20:0	0.68	0.006
12	γ-Linolenic, C18:3 n-6	1.83	0.022
13	α-Linolenic, C18:3 n-3	16.19	0.035
14	Stearidonic, C18:4 n-3	0.47	0.001
15	Eicosadienoic, C20:2 n-6	0.05	0.001
16	Behenic, C22:0	0.29	0.003
17	Lignoceric, C24:0	0.12	0.006
	Σ-SFA	10.44	0.010
	Σ-MUFA	14.26	0.028
	Σ-PUFA	75.30	0.038

**Table 2:** Assignments of main-band wavenumbers recognized in the spectra of HSO.

Number	Wavenumber (cm^−1^)	Assignment
1	4602	C-H C=C str. combination
2	4667	=C-H str + C=C str. combination *cis*
3	4700	C-H bending C=O stretch. combination
4	5180	3v C=O second overtone
5	5269	2v + 3v combination water
6	5678	2v CH_2_ sym methylenic C-H
7	5790	2v CH_2_ sym methylenic C-H
8	5892	2v CH_3_ asym methylic C-H
9	5963	2v C-H stretching close to *cis* C=C
10	7070	methylenic C-H combination .CH_2_
11	7190	methylenic C-H combination .CH_2_
12	7260	methylic C-H 2v + δ combination .CH_3_
13	8260	3v CH_2_ methylenic C-H
14	8395	3v CH_3_ methylic C-H
15	8580	C=O carbonyl from aliphatic hydrocarbons

**Table 3:** Table of effects of the ASCA model. PCs: principal components; Cum Eigen Val: cumulative eigenvalue.

Model Effect	PCs	Cum Eigen Val	Effect %	*p*-Value
Temperature (temp)	1	0	4.47	0.0002
Time (time)	8	0.03	27.99	0.0002
Storage material (mat)	3	0	3.45	0.0346
Temp × time	8	0.02	17.61	0.0002
Temp × mat	3	0.01	5.34	0.0002
Mat × time	20	0.02	17.06	0.0002
Mean	-	-	0	-
Residuals	-	-	24.08	-

**Table 4:** Results of the calibration model for peroxides, dienes (K_232_), and trienes (K_268_). LVs: latent variables; Rmsec: root-mean-square error of calibration; Rmsecv: root-mean-square error of cross-validation; R^2^ cal: coefficient of determination for calibration; R^2^cv: coefficient of determination for cross-validation.

Model	Mean	Std Dev.	Range		LV	Rmsec	Rmsecv	R^2^ Cal	Bias	R^2^ Cv	RPD
			Min	Max							
Peroxides	5.81	7.60	0.81	44.6	8	1.50	2.50	0.96	0.70	0.71	3.04
Dienes (K_232_)	3.80	1.10	3.14	10.50	7	0.42	0.57	0.82	−0.04	0.73	1.51
Trienes (K_268_)	1.29	0.22	1.11	2.70	5.00	0.06	0.12	0.89	0.05	0.70	1.83

**Table 5:** Matrix of the experimental design.

Temperature (T °C)	Material	Storage Period	Days
T10 (dark) T25 (diffused light)	Polypropylene (PP) Clear glass (CG) Amber glass (AG) Amber glass with aluminum foil (AGA)	1	15
		2	30
		3	45
		4	60
		5	90
		6	120
		7	150
		8	190
		9	270
